# Management and Outcome of Traumatic Intracerebral Hemorrhage in 79 Infants and Children from a Single Level 1 Trauma Center

**DOI:** 10.3390/children8100854

**Published:** 2021-09-26

**Authors:** Harald Binder, Marek Majdan, Johannes Leitgeb, Stephan Payr, Robert Breuer, Stefan Hajdu, Thomas M. Tiefenboeck

**Affiliations:** 1 Department of Trauma Surgery, Medical University of Vienna, 1090 Vienna, Austria; harald.binder@meduniwien.ac.at (H.B.); johannes.leitgeb@meduniwien.ac.at (J.L.); stephan.payr@meduniwien.ac.at (S.P.); robert.breuer@meduniwien.ac.at (R.B.); stefan.hajdu@meduniwien.ac.at (S.H.); 2 Institute for Global Health and Epidemiology, Department of Public Health, Trnava University, 91701 Trnava, Slovakia; mmajdan@truni.sk

**Keywords:** traumatic brain injury, intracerebral hemorrhage, infants, children and adolescent, prognostic factors, outcome

## Abstract

Objective: Traumatic brain injury is a leading form of pediatric trauma and a frequent cause of mortality and acquired neurological impairment in children. The aim of this study was to present the severity and outcomes of traumatic intracerebral bleeding in children and adolescence. Methods: Seventy-nine infants and children with intracerebral bleedings were treated between 1992 and 2020 at a single level 1 trauma center. Data regarding accident, treatment and outcomes were collected retrospectively. The Glasgow Outcome Scale was used to classify the outcome at hospital discharge and at follow-up visits. CT scans of the brain were classified according to the Rotterdam score. Results: In total, 41 (52%) patients with intracerebral bleedings were treated surgically, and 38 (48%) patients were treated conservatively; in 15% of the included patients, delayed surgery was necessary. Patients presenting multiple trauma (*p* < 0.04), higher ISS (*p* < 0.01), poor initial neurological status (*p* < 0.001) and a higher Rotterdamscore (*p* = 0.038) were significantly more often treated surgically. Eighty-three percent of patients were able to leave the hospital, and out of these patients, about 60% showed good recovery at the latest follow-up visit. Overall, 11 patients (14%) died. Conclusion: The findings in this study verified intracerebral bleeding as a rare but serious condition. Patients presenting with multiple traumas, higher initial ISS, poor initial neurological status and a higher Rotterdamscore were more likely treated by surgery. Trial registration: (researchregistry 2686).

## 1. Introduction

Traumatic brain injury (TBI) is a leading form of pediatric trauma and a common cause of mortality and acquired neurological impairment in children [1]. Acute intracerebral bleedings (ICBs) in children, especially in infants and toddlers, are relatively uncommon, with a reported incidence of 1.4 cases per 100,000 children [2,3]. Despite this fact, it seems that pediatric TBI is rarely investigated, and physicians are often left to rely on their clinical experience when making treatment decisions [4]. Due to its rare and atypical occurrence [2,3], pediatric intracerebral bleeding (ICB) poses a significant challenge for diagnosis and treatment. Furthermore, it is not directly comparable to adult ICBs, which are occurring in up to 8.2% of all traumatic brain injuries (TBIs) [5] and 13 to 35% of severe TBIs [6,7,8]. ICBs in children and adolescent are often associated with brain tumors, congenital heart disease and/ or vascular lesions. Overall, the most common cause of ICB in children representing the highest morbidity and mortality is trauma [9,10]. Early recognition of ICB is a crucial due to the danger of delayed development, cerebral palsy, epilepsy and even death. Mental status changes, headaches, convulsions, focal neurological deficits and vomiting are typical clinical signs of these patients [9]. In 2014, a meta-analysis investigating the association of particular symptoms relative to ICB in pediatric patients with minor head trauma presented that skull fractures, focal neurological deficit, seizure and a Glasgow coma scale (GCS) < 15 significantly correlated with ICB. Prior to this, Hamilton et al. [11] showed a very low incidence of ICB in pediatric patients with minor head trauma and a GCS of 15 emphasizing that the GCS is still an important tool. 

The outcome after traumatic intracranial bleeding depends on different factors such as size and localization of the hemorrhage as well as the clinical status at time of presentation. Furthermore, it is associated with a mortality rate of 7%, which is very similar to the mortality of acute epidural hematoma [2,6] and considerably more favorable than traumatic subdural hematoma, especially when associated with child abuse [12].

Another group of researchers presented a correlation between neuromarkers and GOS scores for brain-derived neurotrophic factors and autoantibodies relative to S100 in severe brain injuries in children [13]. 

Due to the development of diagnostic methods in the last decades, computed tomography (CT) has become a standard device for the prompt and accurate detection of ICB [5,6,7,8]. 

The treatment of ICBs depends on the amount of bleeding and neurological symptoms at the time of admission. Nevertheless, the influence of different invasive procedures such as immediate surgery (decompressive craniectomy or a craniotomy) and intracranial pressure (ICP) catheter monitoring and brain tissue oxygenation pressure (PtiO_2_) upon the final outcome is uncertain [14,15,16].

However, variations in outcome and management make it difficult to clarify aspects affecting the clinical presentation of traumatic ICB in children and infants [5,6,7,8]. 

The aim of this study was to present the severity and outcome of traumatic ICB in children admitted to a level 1 trauma center and to analyze differences between surgical and conservative treatment. 

The hypothesis in this retrospective study is that an adequate treatment of ICBs in children and adolescence may result in good clinical and functional outcomes.

## 2. Methods

### 2.1. Data Collection

This study was performed as a retrospective study including all patients (<16 years) admitted with TBI to a level 1 trauma center (blinded for review) between 1992 and 2020. 

The following data were extracted out of patients’ charts: (1) patients demographics (age, sex); (2) cause of injury; (3) injury severity (Injury Severity Score (ISS), (4) Glasgow Coma Scale (GCS) and additional injuries); (5) CT findings; (6) treatment modalities (surgical vs. conservative, types of surgery and additional treatment); and (7) outcomes (Glasgow Outcome Scale (GOS)) at discharge and at follow up.

### 2.2. Treatment Procedures

A rapid examination by an emergency physician, which included the documentation of pediatric GCS and pupillary reactivity, was performed in all admitted patients. The preliminary measures are followed by emergency medical treatment and included rapid-sequence intubation, ventilation, treatment of hemorrhage, treatment of associated substantial extra cranial injury and fluid resuscitation if necessary. 

After admission to the hospital, each patient underwent an immediate CT scan and an examination by a trauma team (consisting of anesthesiologists, trauma surgeons and/or neurosurgeons, radiologists and nurses). Depending on the results of the CT scan, patients underwent surgery and/or were admitted to the ICU or pediatric ward for conservative treatment. The conservative management was in accordance to the guidelines for the management of pediatric severe traumatic brain injury [17] and the S2k guidelines AWMF [18]. The main goal is to reduce the ICP by hyperventilation, osmolar therapy (e.g., Mannitol) and 30 degrees of upper body elevation. Additionally, antibiotics in patients with skull fractures and an anticonvulsive treatment in all patients were initiated. 

Surgical treatment was performed by trauma surgeons and neurosurgery in consultation with neurosurgeons for complex cases. Intensive medical treatment has been implemented by anesthesiologists in cooperation with trauma surgeons and neurosurgeons. 

The documentations of pre-hospital parameters and treatments were conducted by paramedics. CT scan findings were interpreted by trauma surgeons as well as neurosurgeons in collaboration with radiologists specialized in trauma diagnostics. In all patients with bleeding, the Rotterdam Score was calculated [19]. Data regarding the duration of various treatments, complications and outcomes were collected at hospital discharge and at follow up. 

The follow-up protocol after TBI with bleeding conservatively managed includes: IMC stay or admission to the pediatric ward and routine clinical investigations for the first 24 h including monitoring and a neurological evaluation every two to four hours. During the following days, if no neurologic impairment appears, the monitoring process was concluded, and the clinical investigation is extended to every six to eight hours. Follow-up CT scans in conservatively managed patients are only performed by neurologic impairment. 

The follow-up protocol after TBI with bleeding operatively managed includes: ICU stay for at least 48 h and a treatment according to the guidelines for the management of pediatric severe traumatic brain injury [17]. A follow-up CT scan was routinely performed in the first 48 h after surgery. 

After hospital discharge, the patients were asked to attend follow up visits 2 weeks after discharge and then every half year for the first year. Further follow-up visits were planned in accordance to the symptoms presented.

### 2.3. Data Analysis 

The aim of this study was to present characteristics and outcomes in surgically and conservatively treated patients after ICB. Affected patients who underwent at least one cranial surgery during their hospital stay were assigned to the “operated” group; if not, they were assigned to the “conservative” group. Comparisons of demographic factors, injury causes, trauma characteristics, severity and outcome were made between both groups. In the case of continuous variables, medians with respective interquartile ranges were calculated and used as central measures. In the case of categorical variables, the total values with corresponding percentages were calculated as measures of frequency. In order to estimate the population proportions, 95% confidence intervals were calculated wherever percentages were used. All analyses were performed by using the R project statistical environment. A *p*-value of <0.05 was considered statistically significant.

## 3. Results 

Overall, 79 patients were included with the diagnosis.

Demographic factors and injury causes are presented in Table 1. The median age of the patients was 8 years (10 years in the operated and 8.5 years in the conservative group); the male sex was somewhat more prevalent in the “surgically treated” group (66%) and slightly higher in the “conservatively treated” group (55%). The following major causes of injury with respect to their frequency were identified: traffic accidents, falls from over 150 cm height followed by falls from 50 to 150 cm height and sporting accidents. Falls from heights lower than 50 cm as well as children with battered child syndrome were evenly distributed. Falls from over 150 cm were more prevalent in the operated group. Neither acute nor delayed surgical intervention was necessary in the group of falls from less than 50 cm. The distributions concerning falls from 50 to 150 cm were quite similar in all treatment groups (with two patients (5%) in the “conservative group” and one patient (3%) in the “operative group” as well as one patient (8%) in the “delayed surgery group”) (Table 1). An increasing number of high impact traumas with increasing age could be detected, therefore suggesting an association (Table 1 and Table 2). Symptoms including external swellings within the head region (49%), vomiting (15%), impaired consciousness (28%) and severe neurological deficits including coma (51%) are shown in Table 2. In all patients who underwent a surgical procedure, a pathologic neurological status was present, underlining the severity of these traumatic lesions.

**Figure 1:**
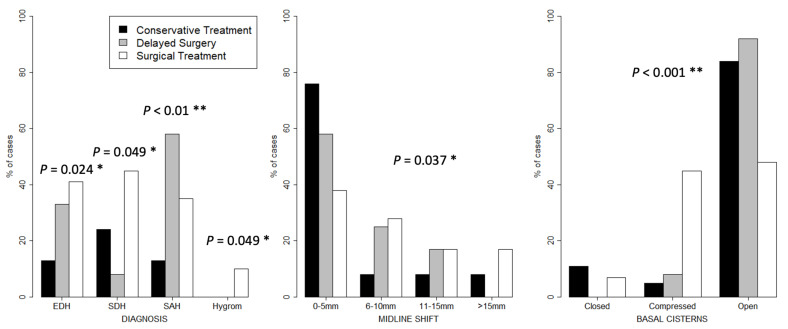
Differences in conservative versus delayed surgical versus operative treatment regarding diagnosis, midline shift and compression of the basal cisterns in relation to patient number in percentages. * = significant; ** = highly significant.

Table 2 also presents injury severity patterns. All operated patients had additional severe injuries. Seventy-nine percent of patients were comatose and their ISS was significantly higher (*p* < 0.01) compared to the conservatively treated group (medians of 25 vs. 16). Fifty-nine percent of the patients in the “operated” group had a Rotterdam CT score of four or higher. In the “conservative” group, a score of ≥4 could be observed in 21%. CT findings (presence of additional diagnoses, midline shift and compression of basal cisterns) are shown in Figure 1, suggesting more severe injuries in the operative group. Treatment factors are shown in Table 3. 

Surgically treated patients showed a higher prevalence (59%) of air transport, and they were significantly more often intubated in the field (79% vs. 32%, *p* < 0.001) and spent also a significantly longer period of time in the intensive care unit (ICU) (21% of the operated group stayed longer than 30 days vs. 5% in the conservative group, *p* < 0.001), emphasizing the severity of traumatic lesions in this group (Table 3). Twenty-three patients (76%) were operated within 1 h after admission, seven patients (24%) underwent two surgical interventions, two patients (7%) had three surgical procedures and one patient (3%) even had four surgical interventions (Table 4). Delayed surgery of ICB was performed on patients with other life-threatening injuries (Table 2) or when conservative management had failed (Table 4). Intracranial pressure (ICP) was monitored in 83% of patients (Table 4). ICP monitoring was removed according to protocol when ICP parameters showed no value > 10 mmHg in 24 h in order to minimize the risk of postoperative infections.

The GOS at hospital discharge showed a significant difference (*p* < 0.001) between the two groups; however, at the latest follow up (mean 12 months; median 6 months), this difference was no longer present (Table 5). The majority of the patients presented favorable outcomes (moderate disability or good recovery) both at discharge (57 patients; 73%) and at follow up (46 patients; 58%). The proportion of unfavorable outcomes (death, vegetative state and severe disability) was higher in the operative group, especially upon hospital discharge. There was a significant difference (*p* < 0.001) concerning the time of death between the conservatively and surgically treated groups. In the surgically treated group, six patients (20%) died, three (10%) within 24 h and two (7%) within 48 h. Fourteen days after delayed surgery, one patient (8%) died due to cardiovascular failure. Another patient (3%) died 21 days after the surgical procedure due to multiorgan failure after sepsis. In the conservatively treated group, three patients (8%) died within 24 h and one (3%) after 48 h past hospital admission. The overall mortality rate of ICBs in infants and children in the sample was 14%. 

In 46 patients (58%), educational achievements could be evaluated at the latest follow-up visit showing that academic performances were hardly affected. Patients who underwent surgery tended to have more unfavorable outcomes with respect to their neurocognitive functions.

## 4. Discussion

This study analyzed the demographic factors, injury severity, treatment and outcomes of 79 cases of pediatric TBI with acute ICB. This study focused on traumatic ICB by comparing surgically and conservatively treated patients. In the current literature, most studies only analyze spontaneous ICB [20,21,22,23]. Studies concerning traumatic etiology such as the Kang et al. [24] or Mandera et al. series [5] are rather rare.

Low GCS scores, high ISS, unfavorable CT findings such as a midline shift of more than 5 mm, compressed or closed basal cisterns or cerebral oedema and neurological deficits were treated rather operatively. These findings did not match the results of Kang et al. [24], presenting a higher incidence of conservative treatment (71%) in their study, whereas Meyer-Heim and Boltshausers reported that 32% of their patients were treated conservatively and 68% were treated surgically [20]. Although there is a difference concerning selection criteria in both studies, Meyer-Heim and Boltshauser et al. [20] included spontaneous ICB, whereas Kang et al. [24] focused on traumatic etiology; still, the above described criteria such as low GCS scores and unfavorable CT findings are applicable in both studies.

Overall, 41 patients (52%) achieved good recovery at the final follow up (Table 5), showing similar findings to Ruf et al. [25]. Mahanna et al. proclaimed no surgical impact on the survival of patients with TBI, but a considerable change concerning neurological progression was reported [26]. These findings did not match with the present study showing a less favorable outcome in the operatively treated group (Table 5). The majority of pediatric series evaluating the benefit of surgical intervention after TBI agree on early surgery (within 48 h after the trauma) [27,28,29]. Delayed surgery was necessary in 15% of our patient collective. As presented in literature, it is of essential importance to treat such patients in order to avoid further damage to the brain [30]. In addition, an increase in different blood parameters may be an early sign of diffuse axonal injury after traumatic brain injury [31].

Moreover, different predictive parameters are presented in the literature, and there are still controversies with respect to the application of surgery or conservative treatment in pediatric ICB [32,33,34]. Another aspect could potentially help such decisions: While in adult head-trauma patients, secondary risk factors influence the outcome significantly [32], such an effect is less apparent in children [4], which is also supported by this study. Furthermore, the combination of higher plasticity and deformity, whereby external forces are absorbed in a different manner compared to adults, should be considered. For example, the lower rigidity of the infant skull as well as open sutures function as joints, allowing a small degree of movement in response to a mechanical stress [35].

In our study, CT findings (midline shift and patency of basal cisterns), GCS score and presence of neurological defects were reliable indicators for surgical treatment. As above mentioned, the percentage of unfavorable outcome (death, vegetative state and severe disability) was higher in the operative group. Caroli et al. [36] and Lobato et al. [37] described unfavorable outcomes in cases with ICB associated with SDH, especially in operatively treated patients. This corresponds with our data. In the present study, SDH was detected in 45% of operated patients and 24% in the conservative group. Furthermore, 35% of operatively treated SAH vs. 13% conservatively treated could be detected. Epidural hematoma (EDH) was shown in 41% of our operated patients and in 13% of the conservatively treated patients. In the operated group, a hygroma could be detected in 10% of the patients (Figure 1). Therefore, these results are assumed to be negative prognostic factors concerning the survival and also according to Bullock et al. [38]. The trend to manage patients with combined lesions conservatively is shown in Figure 1. 

Patients without neurological symptoms, midline-shift less than 5 mm and open basal cisterns may be managed conservatively, showing good neurological outcomes. This is in accordance with the findings in other similar cohorts [4,38]. The hospital mortality of 14% in this study is well in line with the published literature [39,40,41,42,43]. Pulmonary failure and multiorgan failure due to sepsis were reasons for death in two operated polytraumatized children. Multiple injured patients, multiorgan failure due to sepsis and cerebral herniation syndrome were the reasons for death. Diffuse cerebral swelling on initial cranial CT imaging combined with an infaust massive cerebral hemorrhage was the reason for palliative non-surgical treatment in two cases (5%) of acute brain death. Both head injuries were due to child abuse. While subdural hematoma is observed as one of the most common manifestations of abused children [2,44,45,46,47,48], often in combination with subarachnoid hemorrhage (49), ICB is rather rare, probably due to the more elastic vessels. They also tend not to tear as readily than compared to adjacent axons on tissue deformation [48]. This is also confirmed by this present study where only two cases (3%) could be detected. All of them were under the age of 6 years and conservatively managed. Although only 3% of our patients suffered from ICB due to child abuse, the absence of a history of significant accidental trauma mandates a full investigation of maltreatment, which is also recommended in the literature [49]. The above-mentioned higher plasticities and deformity as well as the substantially lower secondary risk factors compared to adults could be the causes for the rarity of this pathology in children when taken together. To summarize, negative prognostic factors for survival include surgically treated ICB combined with additional intracranial lesions (Figure 1), the presence of a polytrauma and diffuse swelling as a potent precursor of cerebral herniation. Although it is very infrequent and, therefore, rare, ICBs resulting from child abuse can also be observed as negative prognostic factors.

There are limitations to this study: It has a retrospective design and a relatively small cohort of patients, which is a consequence of the rather rare occurrence of these specific types of traumas, but nevertheless it limits the generalizability of the findings. There were no homogenous guidelines for treatment in the analyzed center, and treatment decisions were made by the surgeon. However, our study represents a homogenous group and covers the main outcome parameters for a valid conclusion.

Conclusion: Our study confirms that pediatric ICB is a rare but serious condition. In defiance of an overall mortality rate of 14%, 60% of the surviving patients showed no neurological impairment at the latest follow up. Patients presenting with multiple traumas, higher initial ISS, poor initial neurological status and a higher Rotterdamscore were more likely treated by surgery.

**Figure 2:**
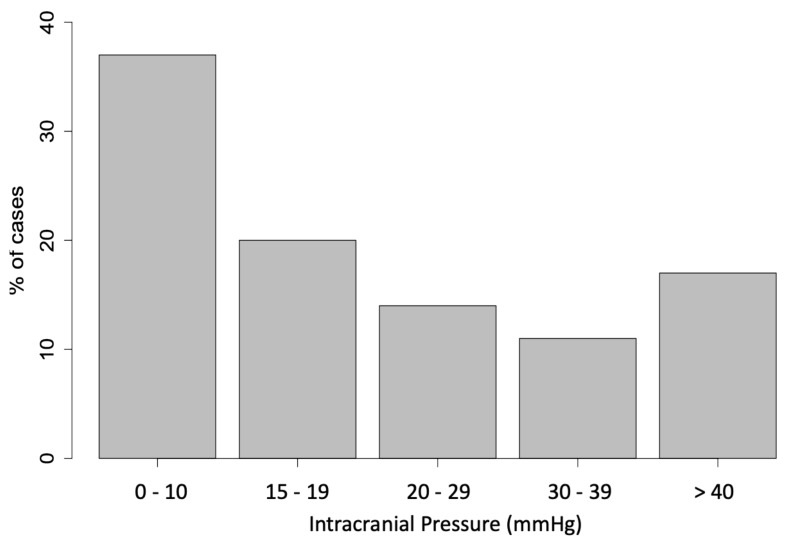
Detailed overview of intracranial pressure (in millimeter of mercury) measured at time of surgery.

Figure legend: Figure 1 represents differences in conservative versus surgical treatment regarding diagnosis, midline shift and compression of the basal cisterns in relation to patient number in percentages. Figure 2 shows a detailed overview of intracranial pressure (in millimeter of mercury) measured at time of surgery.

## Figures and Tables

**Table 1:** Demographic characteristics of patients and characteristics of trauma in conservatively and surgically managed pediatric TBI patients with ICB.

Measure	Conservative	Delayed Surgery	Operative	Total	*p*-Value
Total N (%)	38 (48%)	12 (15%)	29 (37%)	79	
Age (median, IQR)	8.5 (6–13)	7.5 (2.75–11.75)	10 (2–14)	8 (3.5–14)	0.874
Gender (N, % male)	21 (55%)	7 (58%)	19 (66%)	47 (60%)	0.695
Trauma mechanism (N, %)	/	/	/	/	/
Brawl	0	0	1 (3%)	1 (1%)	0.629
Fall (50–150 cm)	2 (5%)	1 (8%)	1 (3%)	4 (5%)	
Fall (<50 cm)	2 (5%)	0	0	2 (3%)	
Fall (>150 cm)	6 (16%)	4 (33%)	11 (38%)	21 (26%)	/
Traffic accident	23 (61%)	7 (58%)	15 (52%)	45 (57%)	/
Sports	2 (5%)	0	1 (3%)	3 (4%)	
Battered child syndrome	2 (5%)	0	0	2 (3%)	
Other	1 (3%)	0	0	1 (1%)	
Multiple trauma (N, % Yes)	24 (63%)	9 (75%)	26 (90%)	59 (75%)	0.04 *

N = number, IQR = interquartile range; * = significant.

**Table 2:** Injury severity indicators in conservatively and surgically managed pediatric TBI patients with ICB.

Measure/Treatment Type	Conservative	Delayed Surgery	Operative	Total	*p*-Value
Total N (%)	38 (48%)	12 (15%)	29 (37%)	79	
Symptoms indicating TBI (N, % Yes)	/	/	/	/	/
External Swellings	19 (50%)	8 (67%)	12 (41%)	39 (49%)	0.335
Nausea	6 (16%)	2 (17%)	1 (3%)	9 (11%)	<0.01
Vomiting	5 (13%)	3 (25%)	4 (14%)	12 (15%)	0.41
Unconsciousness (N, % present)	17 (45%)	7 (58%)	21 (72%)	45 (57%)	0.22
Neurological status (N, %)	/	/	/	/	/
Normal	15 (40%)	0	1 (3%)	16 (20%)	<0.001 **
Somnolent	12 (32%)	5 (42%)	5 (17%)	22 (28%)	
Comatose	11 (29%)	6 (50%)	23 (79%)	40 (51%)	
Pupils (N, %)	/	/	/	/	/
Both reactive	28 (74%)	8 (67%)	17 (59%)	53 (67%)	0.679
One reactive	4 (11%)	1 (8%)	3 (10%)	8 (10%)	
None reactive	6 (16%)	3 (25%)	9 (31%)	18 (23%)	
ISS (median, IQR)	16 (9–21)	25 (17–30)	24 (17–29)	20 (15–26)	<0.01 **
First pediatric GCS (median, IQR)	8.5 (6–13)	7.5 (3–12)	10 (2–14)	8 (3.5–14)	0.869
Admission pediatric GCS (median, IQR)	8.5 (6–13)	7.5 (3–12)	10 (2–14)	8 (3.5–14)	0.869
Vertebral Fracture (N, %)	/	/	/	/	/
Neck Region	4 (11%)	1 (8%)	1 (3%)	6 (8%)	0.553
Thorax Region	2 (5%)	0	1 (3%)	3 (4%)	0.7
Additional Injuries (N, %)	/	/	/	/	/
Upper extremity Fracture	6 (16%)	2 (17%)	3 (10%)	11 (14%)	0.779
Lower Extremity Fracture	5 (13%)	2 (17%)	5 (17%)	12 (15%)	0.824
Injury to thoracic region	8 (21%)	4 (33%)	11 (38%)	23 (29%)	0.18
Injury to abdominal region	5 (13%)	2 (17%)	5 (17%)	12 (15%)	0.888
Rotterdam CT Score (N, %)	/	/	/	/	/
1	0	0	0	0	0.038 *
2	6 (16%)	3 (25%)	3 (10%)	12 (15%)	
3	24 (63%)	7 (58%)	9 (31%)	40 (51%)	
4	2 (5%)	2 (17%)	8 (28%)	12 (15%)	
5	3 (8%)	0	7 (24%)	10 (13%)	
6	3 (8%)	0	2 (7%)	5 (6%)	

CT = computed tomography; GCS = Glasgow coma scale; IQR = interquartile range; ISS = injury severity score; N = number; TBI = traumatic brain injury; ** = highly significant; * = significant.

**Table 3:** Treatment factors in conservatively and surgically managed pediatric TBI patients with ICB.

Measure/Treatment Type	Conservative	Delayed Surgery	Operative	Total	*p*-Value
Total N (%)	38 (48%)	12 (15%)	29 (37%)	79	
Transport (N, % Air)	11 (29%)	5 (42%)	17 (59%)	33 (42%)	0.142
Intubation (N, %)	12 (32%)	7 (58%)	23 (79%)	42 (53%)	<0.001 **
X-Ray performed (N, %)	27 (71%)	10 (83%)	25 (86%)	62 (79%)	0.296
CT scan performed (N, %)	38 (100%)	12 (100%)	29 (100%)	79 (100%)	1
MR performed (N, %)	7 (18%)	3 (25%)	7 (24%)	17 (22%)	0.811
ICU days (N, %)	/	/	/	/	/
No ICU admission	22 (58%)	0	0	22 (28%)	<0.001 **
≤10 days	11 (29%)	4 (33%)	12 (41%)	27 (34%)	
11–20 days	1 (3%)	3 (25%)	5 (17%)	9 (11%)	
21–30 days	3 (8%)	3 (25%)	6 (21%)	12 (15%)	
Over 30 days	2 (5%)	1 (8%)	6 (21%)	9 (11%)	

CT = computed tomography; ICU = intermediate care unit; MR = magnetic resonance; N = number; ** = highly significant.

**Table 4:** Treatment factors in surgically managed pediatric TBI patients with ICB.

Measure	Delayed Surgery	Operative	Total	*p*-Value
/	12 (29%)	29 (71%)	41	
Surgery time (N, %)	/	/	/	/
<1 h	0	23 (100%)	23 (76%)	<0.001 **
<24 h	12 (100%)	0	12 (29%)	
<1 week	1 (8%)	0	1 (2%)	
<2 weeks	1 (8%)	0	1 (2%)	
Delayed after 4 h	2 (17%)	0	2 (5%)	
TBI surgeries within 24 h (N, %)	/	/	/	/
One	9 (75%)	28 (97%)	37 (90%)	0.067
Two	1 (8%)	1 (3%)	2 (5%)	
None	2 (17%)	0	2 (5%)	
Overall number of TBI surgeries (N, %)	/	/	/	/
One	9 (75%)	19 (66%)	28 (68%)	0.9
Two	2 (17%)	7 (24%)	9 (22%)	
Three	1 (8%)	2 (7%)	3 (7%)	
Four	0	1 (3%)	1 (2%)	
Other Surgery (N, %)	/	/	/	/
Multiple surgery	0	3 (10%)	3 (7%)	0.792
Osteosynthesis (external)	2 (17%)	2 (7%)	4 (10%)	
Osteosynthesis (internal)	1 (8%)	2 (7%)	3 (7%)	
Mediastinal drain	0	1 (3%)	1 (2%)	
Other	1 (8%)	2 (7%)	3 (7%)	
None	8 (67%)	18 (62%)	26 (63%)	
Parenchymal ICP monitor (N, %)	9 (75%)	25 (86%)	34 (83%)	0.397
Ventricular drain (N, %)	5 (42%)	4 (14%)	9 (22%)	0.092
Days of ICP monitoring (N, %)	/	/	/	/
≤10 days	5 (41%)	16 (55%)	21 (51%)	0.323
11–20 days	2 (17%)	4 (14%)	7 (17%)	
21–30 days	2 (17%)	4 (14%)	6 (15%)	
31+ days	1 (8%)	2 (7%)	3 (7%)	
None	2 (17%)	3 (10%)	5 (12%)	

CI = confidence interval; ICP = intracranial pressure; N = number; TBI = traumatic brain injury; ** = highly significant.

**Table 5:** Outcomes in conservatively and surgically managed pediatric TBI patients with ICB.

Measure/Treatment Type	Conservative	Delayed Surgery	Operative	Total
	38 (48%)	12 (15%)	29 (37%)	79
GOS–hospital discharge (N, %)	/	/	/	/
Death	4 (11%)	1 (8%)	6 (21%)	11 (14%)
Vegetative state	0	0	1 (3%)	1 (3%)
Severe disability	2 (5%)	3 (25%)	3 (10%)	8 (10%)
Moderate disability	3 (8%)	2 (17%)	12 (41%)	17 (22%)
Good recovery	28 (74%)	6 (50%)	6 (21%)	40 (51%)
Unknown	1 (3%)	0	1 (3%)	2 (3%)
GOS–follow up (N, %)	/	/	/	/
Death	4 (11%)	1 (8%)	6 (21%)	11 (14%)
Vegetative state	0	0	1 (3%)	1 (1%)
Severe disability	0	1 (8%)	1 (3%)	2 (3%)
Moderate disability	1 (3%)	1 (8%)	3 (10%)	5 (6%)
Good recovery	22 (58%)	6 (50%)	13 (45%)	41 (52%)
Unknown	11 (29%)	3 (25%)	5 (17%)	19 (24%)
Time of Death (N, %)	/	/	/	/
Survivors	34 (90%)	11 (92%)	23 (79%)	68 (86%)
Within 24 h	3 (8%)	0	3 (10%)	6 (8%)
Within 48 h	1 (3%)	0	2 (7%)	3 (4%)
Within 14 days	0	1 (8%)	0	1 (1%)
Within 21 days	0	0	1 (3%)	1 (1%)
Cause of death (N, %)	/	/	/	/
Survivors	34 (90%)	11 (92%)	23 (79%)	68 (86%)
Brain death	2 (5%)	0	4 (14%)	6 (8%)
Cardiovascular failure	2 (5%)	1 (8%)	0	2 (4%)
Respiratory/Pulmonary failure	0	0	1 (3%)	1 (1%)
Multiorgan failure (sepsis)	0	0	1 (3%)	1 (1%)

GOS = Glasgow outcome scale; N = number.

## Data Availability

The data presented in this study are available on request from the corresponding author. The data are not publicly available due to privacy.

## Abbreviations

AIS	Abbreviated Injury Scale
CA	Child Abuse
CT	Computed Tomography
EDH	Epidural Hematoma
SDH	Subdural Hematoma
GCS	Glasgow Coma Scale
ICP	Intracranial Pressure
ICU	Intermediate Care Unit
ISS	Injury Severity Score
ICB	Intracerebral Bleeding
TBI	Traumatic Brain Injury

## STROBE Guidelines

To be used by authors of all observational clinical studies published in OTSR. For this purpose, a cohort study (the term used by STROBE) is considered a longitudinal study typically reporting outcomes of treatment in one or more cohorts; a case-control study is one that identifies the factors in outcomes; a cross-sectional study is one that identifies the prevalence of factors or characteristics in a population at a single point in time.

This checklist table is modified from The STROBE Initiative, www.strobe-statement.org, access date 15 January 2021 and should be filled and submitted within the electronic submission.

	**Item No**	**Recommendation**	**Please Insert Check Where Included or N/A Where Not Applicable**
**Title and abstract**	1	(*a*) Indicate the study’s design with a commonly used term in the title or the abstract	check
		(*b*) Provide, in the abstract, an informative and balanced summary of what was performed and what was found	check
**Introduction**			
Background/rationale	2	Explain the scientific background and rationale for the investigation being reported	check
Objectives	3	State specific objectives, including any pre specified hypotheses	check
**Methods**			
Study design	4	Present key elements of study design early in the paper	check
Setting	5	Describe the setting, locations and relevant dates, including periods of recruitment, treatment, follow up and data collection	check
Participants	6	(*a*) *Cohort study*—Provide the eligibility criteria and the sources and methods of selection of participants. Describe methods of follow up *Case-control study*—Provide the eligibility criteria and the sources and methods of case ascertainment and control selection. Provide the rationale for the choice of cases and controls *Cross-sectional study*—Provide the eligibility criteria and the sources and methods of selection of participants	check
		(*b*) *Cohort study*—For matched studies, provide matching criteria and number of treated and untreated *Case-control study*—For matched studies, provide matching criteria and the number of controls per case	
Variables	7	Clearly define all outcomes, exposures, predictors, potential confounders and effect modifiers. Provide diagnostic criteria, if applicable	check
Data sources/measurement	8 *	For each variable of interest, provide sources of data and details of methods of assessment (measurement) Describe comparability of assessment methods if there is more than one group	check
Bias	9	Describe any efforts to address potential sources of bias	check
Study size	10	Explain how the study size was arrived at	check
Quantitative variables	11	Explain how quantitative variables were handled in the analyses. If applicable, describe which groupings were chosen and why	check
Statistical methods	12	(*a*) Describe all statistical methods, including those used to control for confounding factors	check
/		(*b*) Describe any methods used to examine subgroups and interactions	/
/		(*c*) Explain how missing data were addressed	check
/		(*d*) If applicable, explain how loss to follow up was addressed	check
/		(*e*) Describe any sensitivity analyses	/
**Results**			
Participants	13 *	(a) Report numbers of individuals at each stage of study—e.g., numbers potentially eligible, examined for eligibility, confirmed eligible, included in the study, completing follow up and analyzed	check
/		(b) Give reasons for nonparticipation at each stage	check
Descriptive data	14 *	(a) Give characteristics of study participants (e.g., demographic, clinical, social) and information on other treatments and potential confounders	check
/		(b) Indicate number of participants with missing data for each variable of interest	check
/		(c) *Cohort study*—Summarize follow-up time (e.g., average and total amount)	check
Outcome data	15 *	Report numbers of outcome events or summary measures over time	N/A
Main results	16	(*a*) Provide unadjusted estimates and, if applicable, confounder-adjusted estimates and their precision (e.g., 95% confidence interval). Make clear which confounders were adjusted for and why they were included	/
/		(*b*) Report category boundaries when continuous variables were categorized	N/A
/		(*c*) If relevant, consider translating estimates of relative risk into absolute risk for a meaningful time period	N/A
Other analyses	17	Report other analyses performed—e.g., analyses of subgroups and interactions and sensitivity analyses	N/A
**Discussion**			
Key results	18	Summarise key results with reference to study objectives	check
Limitations	19	Discuss limitations of the study, taking into account sources of potential bias or imprecision. Discuss both direction and magnitude of any potential bias	check
Interpretation	20	Provide a cautious overall interpretation of results considering objectives, limitations, multiplicity of analyses, results from similar studies and other relevant evidence	check
Generalisability	21	Discuss the generalisability (external validity) of the study results	check
**Other information**			
Funding	22	Provide the source of funding and the role of the funders for the present study and, if applicable, for the original study on which the present article is based	check
* Provide information separately for cases and controls. Note: An Explanation and Elaboration article discusses each checklist item and provides methodological background and published examples of transparent reporting. Information on the STROBE Initiative is available at http://www.strobe-statement.org, access date 15 January 2021.			
